# Supplementary material for: Causal interplay between lactose intolerance and gut microbiota: a combined bidirectional Mendelian randomization and in vivo validation study
Source: Front Nutr. 2026 Jun 1;13:1803337. doi: 10.3389/fnut.2026.1803337 (PMC13265576; doi:10.3389/fnut.2026.1803337)
Supplement: Supplementary file 1 [file Data_Sheet_1.zip › supplementary materials/Forward/leave one out cross validation/ebi-a-GCST90027493.finngen_R12_E4_LACTONAS.pdf]

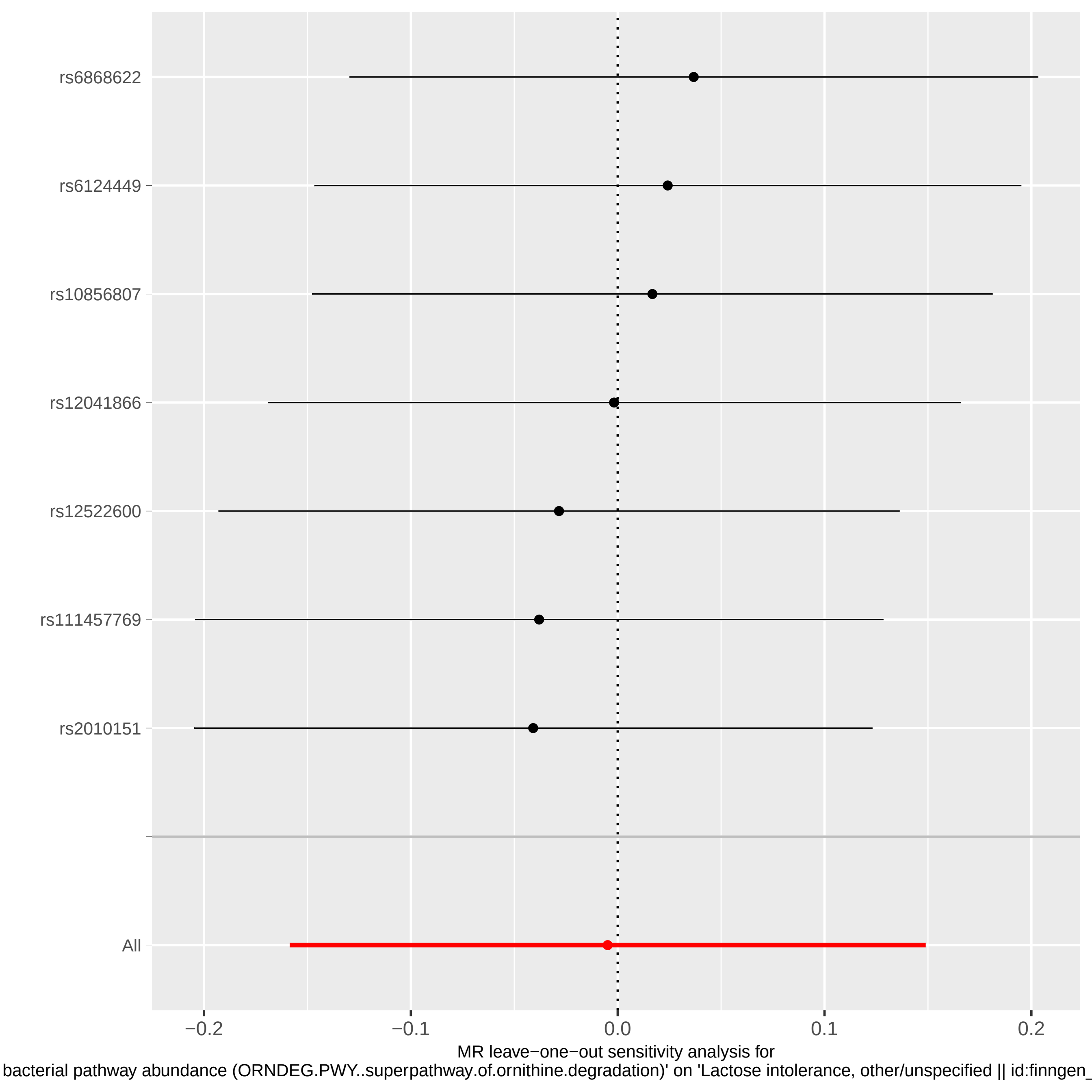

rs6868622
rs6124449
rs10856807
rs12041866
rs12522600
rs111457769
rs2010151
All
−0.2
−0.1
0.0
0.1
0.2
MR leave−one−out sensitivity analysis for
bacterial pathway abundance (ORNDEG.PWY..superpathway.of.ornithine.degradation)' on 'Lactose intolerance, other/unspecified || id:finngen_
